# Current trends in patient and public involvement in cancer research: A systematic review

**DOI:** 10.1111/hex.12841

**Published:** 2018-10-30

**Authors:** Kathrine Hoffmann Pii, Lone Helle Schou, Karin Piil, Mary Jarden

**Affiliations:** ^1^ Institute of Nursing and Nutrition Copehagen University College Copenhagen Denmark; ^2^ Department of Oncology Copenhagen University Hospital Rigshospitalet Copenhagen Denmark; ^3^ Department of Hematology Copenhagen University Hospital, Rigshospitalet Copenhagen Denmark; ^4^ Department of Public Health Faculty of Health and Medical Sciences University of Copenhagen Copenhagen Denmark; ^5^ Department of Public Health Aarhus University Denmark

**Keywords:** cancer research, patient and public involvement, systematic review

## Abstract

**Background:**

Patient and public involvement (PPI) in health research is on the rise worldwide. Within cancer research, PPI ensures that the rapid development of medical and technological opportunities for diagnostics, treatment and care corresponds with the needs and priorities of people affected by cancer. An overview of the experiences, outcomes and quality of recent PPI in cancer research would provide valuable information for future research.

**Objective:**

To describe the current state of PPI in cancer research focusing on the research stages, applied methods, stated purposes and outcomes, and challenges and recommendations.

**Methods:**

A search was conducted on PubMed, CINAHL and PsycINFO for literature published from December 2006 to April 2017. Original research studies describing the involvement of cancer patients, stakeholders and carers as active partners at any stage of the research process were included.

**Results:**

Twenty‐seven studies were included, the majority reporting PPI at the early stages of research, that is, during the definition and prioritization of research topics and the development of recruitment strategies. Few studies reported PPI at later stages and across the research process. Challenges and recommendations were only briefly described, and critical reflection on the PPI process was lacking.

**Conclusion:**

PPI needs to be integrated more broadly in the cancer research process. The quality of reporting PPI should be strengthened through greater critical reflections including both positive and negative experiences of the PPI process. This will contribute to the further development of PPI and its potential in cancer research.

## 1 INTRODUCTION

In the last decades, patient and public involvement (PPI) in health research has steadily grown worldwide.^1^ Various policy directives promoting PPI have been introduced, and funding bodies increasingly require the integration of PPI into research projects.^2^, ^3^ PPI is well established in North America, the UK and Australia through support organizations such as Patient‐Centered Outcomes Research Institute (PCORI) in the United States and INVOLVE in the UK, and through the dissemination of PPI models and scientific publications.^1^, ^4^, ^5^

Involving patients, carers, patient organizations and communities in the research process is valued for multiple reasons. First of all, PPI is related to democratic values as it empowers patients and citizens to influence the research agenda, a task traditionally led by clinicians, researchers and industry.^4^, ^6^, ^7^ The democratizing value of PPI is often described based on various degrees of involvement, in accordance with Arnstein's 1969 ladder of citizen participation,^8^ which ranges from *non‐participation* to *tokenistic involvement* to degrees of *citizen power*.^9^ INVOLVE distinguishes between three PPI approaches: consultation, collaboration and user‐led.^10^ Similarly, Health Canada divides PPI into five stages: inform or educate, gather information, discuss, engage and partner.^11^

Aside from the democratizing value of PPI, it is also valued for potentially enhancing the quality of research. PPI can improve methodological quality, for example, by increasing recruitment and retention of study participants because patient/public representatives have better access to the study population that they are part of, thereby ensuring study acceptability in the target population.^12^ At an epistemological level, proponents of experience‐based knowledge argue that patients and carers’ personal experiences of illness are important contributions to clinical research‐based knowledge.^3^, ^5^

Patient and public involvement methods and approaches cover a broad range of areas,^10^, ^12^, ^13^ such as conventional qualitative and quantitative research methods (eg, interviews, focus groups and surveys), which are applied either independently or combined. Furthermore, PPI also employs approaches and methods related to project management, where patient/public participants are consulted, for example, in Delphi rounds or serve as representatives in steering committees and on expert panels that discuss research design, results and dissemination. PPI is also being practised using more comprehensive models, which include several steps and methods (scientific and non‐scientific). The James Lind Alliance (JLA), for instance, is an independent organization funded by the National Institute for Health Research and the Medical Research Council in the UK and provides a platform for applying an integrative approach which brings patients, carers and health‐care professionals together in Priority Setting Partnerships. The approach uses deliberative methods to identify uncertainties, interpret these as potential research questions and compare these to the existing evidence before engaging in different methods for prioritization (eg, expert panels, surveys, focus groups). Often the final prioritization takes place at face‐to face meetings with group discussions.^7^, ^14^, ^15^ PPI is also an integral aspect of participatory action research^16^ and community‐based participatory research, each of which has its own set of methodologies and approaches.^17^

The variety of values and methods associated with practising PPI make forming an overview and developing recommendations for best practice difficult. With PPI becoming increasingly common in research, the discussion continues regarding its purposes, outcomes and impact, not to mention who it benefits and what quality standards should be applied to evaluate PPI.^18^, ^19^, ^20^, ^21^

### 1.1 PPI in cancer research

Cancer affects a vast population of patients, survivors, relatives and carers. The growing prevalence, uncertain (life‐threatening) prognosis and a high symptom burden make PPI relevant in cancer research to ensure that the rapid development of medical and technological opportunities for diagnostics, treatment and care is aligned with the needs and priorities of the growing population of people affected by cancer. Years of initiatives in the UK and the United States have made PPI a familiar aspect of cancer research, especially due to formal training requirements and the presence of patient advocates/representatives on review panels since the 1990s.^5^ Therefore, PPI in cancer research is a field particularly suitable to study and learn from in terms of how PPI is practised and which outcomes and impact PPI produces.

Earlier reviews in the field include a study by Hubbard et al^5^ that focused on PPI in cancer research, policy, planning and practice from 1994 to 2004. In a later review of 52 research papers, evaluations and recommendations, Hubbard et al^22^ centred on PPI in research. They distinguished between involvement in scientific review panels and participatory research projects (n = 7), and involvement in clinical trials (n = 3). The review showed that PPI in cancer research has been carried out primarily in the United States and the UK, reflecting a general PPI trend. Their results also highlighted that involvement was more prominent in women with breast cancer (n = 22 publications). Moreover, studies mainly reported the impact of PPI on research designs, accrual and response rates. The authors concluded that the agenda of involvement in cancer research has taken root but that evaluation is needed to show the impact of involving patients in the research process.^22^ Because PPI in cancer research continues to grow, gaining an overview of the experiences, challenges, outcomes and quality of more recent PPI in cancer research is an important step in providing information and recommendations for future PPI in cancer research.

The aim of this review is to describe the current state of PPI in cancer research. Three central research questions will be explored: (a) At which stages of research does PPI take place and which methods are applied? (b) What are the stated purposes and outcomes of PPI? and (c) What are the stated challenges and recommendations of the PPI process? The findings from this review are discussed in terms of the democratic and research‐oriented values of PPI in cancer research.

## 2 METHODS

### 2.1 Search strategy

A systematic approach based on the PRISMA guidelines was applied to report the results.^23^ Three databases were systematically searched: MEDLINE/PubMed, the Cumulative Index to Nursing and Allied Health Literature (CINAHL) and PsycINFO. The strategy was customized for each database and included controlled vocabulary, for example, Medical Subject Headings (MeSH terms) and free‐text keywords to identify relevant studies for this review.

The search was conducted using the following keywords: Cancer, Hematol*, Oncolog*,AND Citizen driven, Community participation, Consumer involvement, Consumer participation, Engaging patients, Involving patients, Lay involvement, Lay participation, Partnership*, Partnership, Patient driven, Patient engagement, Patient involvement, Patient participation, Patients view*, Public engagement, Public involvement, Public participation, Stakeholder*, Stakeholder driven, User driven, User involvement, User participation, AND Research agenda*, Design, Priorities, Priority, Prioritization, Prioritizing, Process.

The full search strategy for each database is available upon request by contacting the corresponding author (KHP). The search was exported and managed in RefWorks, including identification of duplicates. The search was limited to the English language and included studies published from December 2006 to April 2017.

### 2.2 Inclusion and exclusion criteria

This review included original research studies describing the involvement of cancer patients, survivors and carers at any stage of the research process with a clear PPI purpose and outcome. Studies that solely described user experiences with involvement in research were excluded if the purpose and outcomes of the PPI in the research process were not described. Other exclusion criteria were studies that described PPI in service development, if no follow‐up research was conducted, prevention and screening projects.

**Figure 1:**
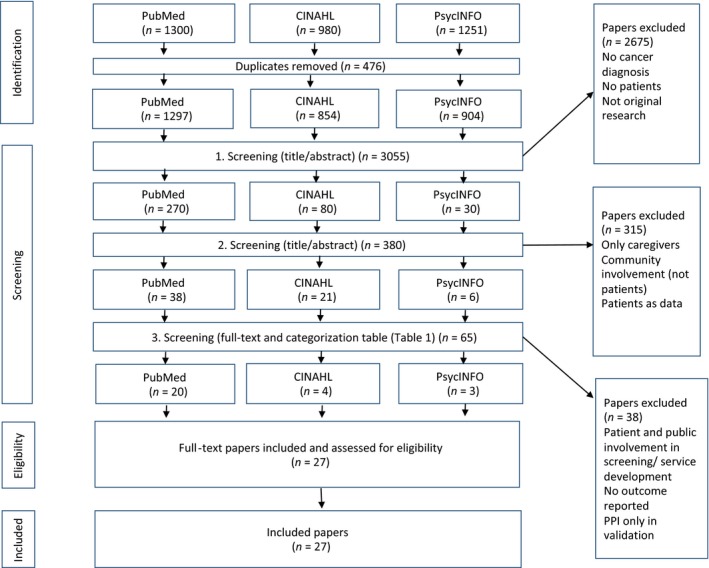
PRISMA flow chart

The first author (KHP) carried out a systematic search in December 2016 and in April 2017 in cooperation with information specialists. After duplicates were removed, the search resulted in 1297 hits in PubMed, 854 in CINAHL and 904 in PsycINFO (Figure 1).

KHP reviewed the titles and/or abstracts in the searches and excluded studies that did not match the inclusion criteria. Two researchers (KHP and LS) then divided the remaining abstracts between them and reviewed the abstracts excluded by the other researcher. If the researchers disagreed, the study was included for full‐text assessment. For the studies initially agreed upon, the researchers did a full‐text reading and assessment. The sorting process resulted in 27 studies whose validity was assessed with the Critical Appraisal Skills Programme (CASP) checklist for qualitative studies and Mixed Method Appraisal Tool (MMAT) for mixed and quantitative studies.^24^ The quality assessment was carried out to gain insight into the methodological quality of the articles but did not result in further exclusions.

### 2.3 Data analysis

Data analysis was conducted by KHP and LS and discussed with KP and MJ in cases of discrepancy. Data regarding PPI methods were extracted according to Table 1, which was developed during the review process. Initially, the table indicated three overall stages: research development, conducting research and research dissemination inspired by similar tables.^4^, ^13^ However, the three stages were further divided into ten subcategories to align with nuances in the included studies (Table 1).

**Table 1:** Research stages of patient and public involvement

Research stage	Subcategories	Definition
1. Development of research focus	Research definition	Definition of research themes/questions
	Research prioritization	Prioritization of research themes/questions
2. Development of research design	Method development	Development of research tools, for example, questionnaires, interview guides, patient‐reported outcome measurements
	Study design development	Development of entire study designs
3. Recruitment	Recruitment strategy	Development of recruitment/retention strategies for research projects
	Recruitment	Participation in recruiting research participants
4. Data generation	Data generation	Participation in data generation, for example, interviewing
5. Data processing	Analysis	Participation in data analysis
6. Research dissemination	Dissemination	Dissemination of research, for example, co‐author/presenter
	Dissemination strategy	Development of dissemination strategies

Furthermore, the following data were extracted according to Table 2: publication year, study origin, population (cancer disease), methods applied, number of involved patients/carers, the stated purpose of PPI, PPI outcomes, the stated challenges specifically related to PPI and the stated recommendations specifically related to PPI.

**Table 2:** Characteristics of included studies stratified according to research stages (cf. Table 1)

Authors, year, origin	Population	Methods	Participants	Purpose	Outcome	Challenges	Recommendations
Research stage: Prioritization of research theme/questionIncluded studies (n = 5)							
Perkins et al,^28^ 2008, UK	Palliative	Questionnaire	Pt (n = 112)	Prioritize 17 research topics	17 topics were prioritized	None	None
		Pilot test	Pt (n = 10)				
McNair et al,^35^ 2016, UK	Bowel cancer (other bowel diseases)	Focus groups	Pt (n = 12) (Total n=NR)	Explore pts’ view on colorectal research and to prioritize research topics with pts and the public	25 research questions were prioritized	None	None
		Interviews	Pt (n = 11) (Total n = 25)				
Moorcraft et al,^26^ 2016, UK	Heterogeneous, mainly breast cancer	Survey	Pt (n = 780)	Prioritize 12 research themes	12 predefined research themes were prioritized	Representativity: Mainly white and middleclass population, who are influenced by ongoing research	None
van Merode et al,^49^ 2016, The Netherlands	Blood cancer	Dialogue Model:		Identify top 10 priorities	Top 10 questions identified and top three stated as research questions	Representativity: Lack of insight from pts with low level of health literacy	None
		Interview	Pt (n = 10)				
		Focus group	Pt (n = 20)				
		Questionnaire	Pt (n = 789)				
		Dialogue meeting/project group	Pt (n = 6)				
Wan et al,^27^ 2016, UK	Endometrial cancer	James Lind Alliance priority setting process:		Identify top 10 unanswered research questions	10 research questions identified	Representativity: Ethnic minorities and +60 y women are under‐represented	None
		Steering group (pts/other stakeholders)	(n = NR)				
		Survey 1	Pt (n = 177) (Total n = 413)				
		Survey 2	Pt (n = NR) (Total n = 113)				
		Consensus meeting	Pt (n = NR) (Total n = 23)				
**Research stage: Definition of research themes/questions** Included studies (n = 5)							
Corner et al,^6^ 2007, UK	Heterogeneous	Focus group	Pt (n = 105)	Reach consensus on research priorities	15 research themes identified and prioritized	Representativity: Few men, ethnic minorities and pts with aggressive tumours included	To compare the priorities to with the views of the public, people bereaved by cancer, and patients in other contexts such as resource poor countries
Perkins et al,^25^ 2007, UK	Palliative	Focus group	Pt (n = 19)	Identify key priorities for future research	5 research themes identified	Maintaining pts focus on research priorities (not their own illness experiences)	Sensitive facilitation when dealing with critically ill participants
							Focus groups are a valid method for developing research ideas
Clinton‐Macharg et al,^43^ 2010, Australia	Haematology	Delphi method	Pt (n = 2)	Develop and prioritize research items	Research items prioritized	Representativity: Under‐representation of newly diagnosed and relapsed pts	The value‐weighting approach represents an acceptable and feasible way to quantify stakeholder perceptions on the allocation of research resources
		Survey (+Pilot test)	Pt (n = 10) (n = NR)				
Saunders et al,^45^ 2012, Australia	Heterogeneous	Workshop	Pt (n = 32)	Identify top 5 cancer research needs	Top 4 research needs identified	None	Build consumer and researcher PPI skills
		Survey	Pt (n = 57)				
Stephens et al,^32^ 2015, UK	Mesothelioma (lung cancer)	James Lind Alliance priority setting partnership/process:		Agree on top 10 interventional research priorities	52 unique unanswered research questions identified Top 10 research questions stated	Pts have short survival, difficult to recruit pts for steering group Pts find medical jargon difficult to understand Pts find prioritization difficult (research issues equally important)	None
		Steering group	Pt (n = 2) Carers (n = 2)				
		Survey	Pt (n = 103) Carers (n = 242)				
		Interim prioritization survey	Pt (n = 38) Carers (n = 98)				
		Consensus meeting	Pt (n = 6) Carers (n = 4)				
**Research stage: Development of recruitment/retention strategies for research projects** Included studies (n = 7)							
Dellson et al,^47^ 2010, Sweden	Breast cancer	Focus group	Pt (n = 5)	Gain insight to pts’ opinions about clinical trial information material	Recommendations for clinical trial information material Pts give new insights, simple improvements that may increase readability/recruitment	Representativity: Informants already active in cancer association Less proactive informants not represented	None
		Questionnaire (validation)	Pt (n = 18)				
Dear et al,^44^ 2011, Australia	Heterogeneous	Consumer reference group discussions	Consumers (n = 11)	Develop user‐friendly clinical trial website	Consumer input is implemented in design of website	None	Working with well‐established consumer networks and projects that already are consumer priorities Researchers listen/respond to consumer needs Researchers support consumer groups
		Survey(evaluation)	Pt (n = 47)		Survey (evaluation): 89% of 47 pts rated the website as good		
Ashley et al,^29^ 2012, UK	Heterogeneous (breast, colorectal, prostate)	Individual and group interviews	Pt (n = 15)	Define best time for recruiting pts for PROM‐based research (psychosocial)	Preferable time found	Representativity: Sample did not include ethnic minorities, and people with advanced cancer disease	None
			HP (n = 15)				
Wells et al,^40^ 2012, US	Heterogeneous	Interview	Pt (n = 18)	Develop clinical trial decision aid	A multi‐media, psycho‐educational intervention for clinical trials	None	None
		Pre‐test	Pt/carers (n = 20)				
Fleisher et al,^31^ 2014, UK	Heterogeneous	Focus groups	Pt/pt advocates (n = 22)	Develop digital decision aid tool to improve preparation for decision making in cancer trials	A high quality, pt‐centred decision aid	Labour intensive and time consuming	None
		Feedback/video development	Pt (n = 5)				
Taylor et al,^34^ 2015, UK	Heterogeneous, young (14‐26 y)	Workshop	Pt (n = 9)	Develop research project brand to increase recruitment and retention	Higher acceptance and retention in study than expected (80% vs 60%)(Lower refusal rate than expected (<20% vs 35%)	None	None
		Survey	Pt (n = 249)				
Taylor et al,^33^ 2016, UK	Heterogeneous, young (14‐26 y)	Workshop (incl. focus groups, individual reflections and creative interpretation)	Pt (n = 8)	Elicit young people's views on access and participation in research to inform recruitment for research project	3 important recruitment themes described	Representativity: Engaging less proactive pts	None
		Survey	Pt (n = 222)				
Research stage: Development of methods							
Included studies (n = 4)							
Vivat et al,^46^ 2012, UK, Germany, Italy, Iceland, Japan, Spain	Palliative cancer	Interview	Pt (n = 22)	Develop cross‐cultural questionnaire on spiritual well‐being among palliative cancer pts	A well‐tested cross‐cultural questionnaire (EORTC QLQ‐SWB36)	Representativity: Few ethnic minorities participated	None
		Survey: Pre‐pilot test	Pt (n = 17)				
		Pilot test	Pt (n = 113)				
McCarrier et al,^41^ 2016, US	Lung cancer	Interviews	Pt (n = 51)	Develop a new symptoms‐based patient‐reported outcome (PRO) instrument	PRO instrument developed according to pt responses and feedback	None	None
		Interviews	Pt (n = 20)				
Treiman et al,^39^ 2016, US	Colorectal cancer	PCORI conceptual model: Advisory board	Pt (n = 7)	Develop and test survey questions	Survey developed according to pt input	None	Involve pts/stakeholders as early as possible Find “common language” to ensure effective communication between researchers and PPI participants Train pts Hold separate meetings with pts Provide remuneration for pts
		Pre‐test: online survey	Pt (n = 23)				
		Interview	Pt (n = 17)				
Sperling et al,^48^ 2016, Denmark	Heterogeneous, adolescents and young adults (17‐38 y)	Interview/focus group	Pt (n = 21)	Develop a new national questionnaire targeting adolescents and young adults with cancer aiming to evaluate treatment and survivorship from the perspective of the pts and to reflect their needs and experiences throughout the cancer trajectory	New questionnaire developed	Representativity: More disadvantaged/ill pts did not participate	None
		Pt panel	Pt (n = 9)				
		Interview	Pt (n = 11)				
Research stage: Development of entire study design Included studies (n = 3)							
Freysteinson,^37^ 2010, US	Breast cancer	Case study/community consultation model	Pt (n = 24) HP (n = 16)	Explore community perspective on legitimacy, benefits, protection and partnership in research idea	Insights into the study design: legitimacy, benefits, protection and partnership A more ethical research design	None	Community consultation model recommended for a more ethical research design
Ellis et al,^30^ 2012, UK	Lung cancer	Interview	Pt (n = 37) Carers (n = 23)	Identify pts and carers views on the desirable components of a novel nonpharmacological intervention	Key issues on development, delivery and uptake of a novel intervention (next step is to pilot test)	None	None
Rush et al,^36^ 2015, US	Breast cancer Latino	Community‐based organization partners Advisory board Feedback	Pt (n = NR)	Develop a culturally sensitive quality‐of‐life survivor‐caregiver intervention (RCT)	Intervention/RCT developed, including survey	None (but implicitly addressed in recommendations/lessons learned)	Lessons learned: Establish trusting/respectful relationships Be receptive to what is already being done in the community Have a plan for addressing questions and conflicts Use democratic approaches for decision making Clear roles and responsibilities Communicate regularly with whole team to promote team cohesion Ensure that study files/procedures are clear and accessible to all collaborators Be prepared to spend time educating team members on study process/design Offer training that meets partner needs Be prepared for higher administrative burden due to large team Be flexible
Multiple research stages: Development of recruitment/retention strategies for research projects, Development of methods, Development of entire study design, Participation in recruitment, Participation in data analysis, Dissemination of research)Included studies (n = 1)							
Chiu et al,^50^ 2013, Canada	Breast cancer	Pilot interviews	Pt (n = 6)	Develop study design (survey package) to increase validity, enhance ethics and gain high response rate	Study design (survey package) developed and validated by pts High survey response rate (81%)	Potential risk of participants rejecting design/method delaying project and increasing the cost	Include cost of care and comfort (support, rest, food) in budgets
		Community advisory group	Pt (n = 5)				
		Workshop	Pt (n = 18)				
		Pt interviews also conducted, but served as data	Pt (n = 46)				
		Survey	Pt (n = 500)				
Multiple research stages: Definition of research themes/questions, Development of recruitment/retention strategies for research projects, Develop dissemination strategy Included studies (n = 1)							
Islam et al,^38^ 2014, US	Lung cancer	Focus groups	Pt (n = 7) (Total n = 36)	Define focus for pt‐centred outcome research (define treatment success) and gain insight into research recruitment/retention and dissemination	Pt‐centred treatment success defined by pts/stakeholders Insights into research recruitment/retention and dissemination	None	None
Multiple research stages: Definition of research themes/questions, Prioritisation of research themes/questions, Development of methods, Dissemination of research, Develop dissemination strategy Studies included (n = 1)							
Vargas et al,^42^ 2014, US	Heterogeneous	CPPR model/community‐academic council Modified Delphi	Pt 28% of 36 members approx. (n = 10)	Form CPPR model to promote cancer research and develop activities/products (eg, education/information) that reduce cancer disparities	CPPR model led to: Conferences (dissemination of research) New survey instrument New research projects Funding Education	Representativity: Under‐representation of male and Latino population (mostly Afro‐American women participated)	Delphi method ensures transparency and equity in development of action Ensure financial support for all partners Ensure time for meetings and planning

CPPR, Community partnered‐participatory research; HPs, health professionals; NR, not reported; Pt, patient (Pts = Patients); PPI, Patient and public involvement; PRO, patient‐reported outcome; PCORI, Patient‐Centered Outcomes Research Institute; RCT, Randomized controlled trial; EORTC QLQ‐SWB36, European Organisation for Research and Treatment of Cancer‐Quality of Life Questionnaire‐Spiritual wellbeing (36 items).

## 3 RESULTS

Based on the review's inclusion criteria, 27 articles were included. Table 2 presents the findings subtracted from the articles. In the following, we describe the current PPI trends and characteristics in cancer research.

### 3.1 Study characteristics: origin and population

The UK represents the majority of the publications (n = 12),^6^, ^25^, ^26^, ^27^, ^28^, ^29^, ^30^, ^31^, ^32^, ^33^, ^34^, ^35^ followed by the United States (n = 7)^36^, ^37^, ^38^, ^39^, ^40^, ^41^, ^42^ and Australia (n = 3).^43^, ^44^, ^45^ A single study represents several countries (Germany, Iceland, Italy, Japan, Spain, the UK),^46^ and the remaining studies report findings from other countries: Sweden,^47^ Denmark,^48^ the Netherlands^49^ and Canada.^50^

The populations in the studies were defined in various ways, though most were disease‐specific, while other populations were defined according to age or ethnicity. The majority of the studies (n = 13) focused on specific cancer diseases: breast cancer (n = 4),^36^, ^37^, ^47^, ^50^ including a study specifically focusing on breast cancer in a Latino population,^36^ followed by lung cancer (n = 4),^30^, ^32^, ^38^, ^41^ blood cancer (n = 2),^43^, ^49^ colorectal cancer (n = 1),^39^ gynaecological cancer (n = 1)^27^ and bowel cancer (and other bowel diseases) (n = 1).^35^ Some studies (n = 8) represented heterogeneous cancer types with no specific population/disease focus.^6^, ^26^, ^29^, ^31^, ^40^, ^42^, ^44^, ^45^ Other studies (n = 8) also represented heterogeneous cancer types but had specific foci, for example, studies on young people (n = 3),^33^, ^34^, ^48^ palliation (n = 3)^25^, ^28^, ^46^ and a community with high cancer disparity (n = 1).^42^

### 3.2 Research stages and applied PPI methods

**Figure 2:**
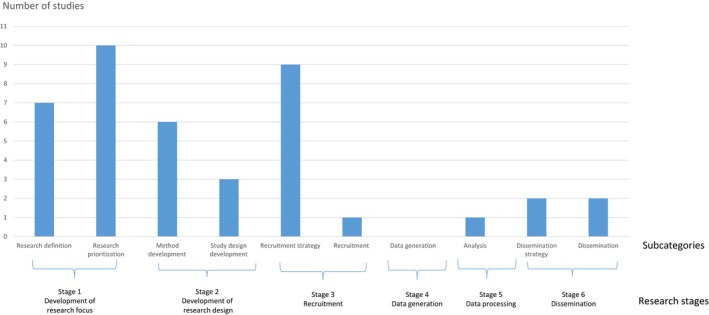
Patient and public involvement at different research stages

Figure 2 shows the distribution of studies for each research stage. The majority of the studies (n = 20) reported PPI at a single stage in the research process. Some studies (n = 5) involved patients at two stages (both the definition and prioritization of research questions/themes).^6^, ^25^, ^32^, ^43^, ^45^ A few studies (n = 3) involved patients/the public at various research stages.^38^, ^42^, ^50^ Because these studies reported PPI at several stages, the number of research stages (n = 41) in the figure exceeds the number of studies included (n = 27). Most studies reported PPI for prioritization of research themes/questions (n = 10),^6^, ^25^, ^26^, ^27^, ^28^, ^32^, ^35^, ^43^, ^45^, ^49^ followed by development of recruitment strategies for research (n = 9),^29^, ^31^, ^33^, ^34^, ^38^, ^40^, ^44^, ^47^, ^50^ definition of research themes/questions (n = 7),^6^, ^25^, ^32^, ^38^, ^42^, ^43^, ^45^ method development (n = 6),^39^, ^41^, ^42^, ^46^, ^48^, ^50^ study design development (n = 3),^30^, ^36^, ^37^ dissemination strategy (n = 2),^38^, ^42^ dissemination (n = 2),^42^, ^50^ analysis (n = 1)^50^ and recruitment (n = 1).^50^ None of the studies involved patients in data generation (eg, as interviewers/facilitators).

**Figure 3:**
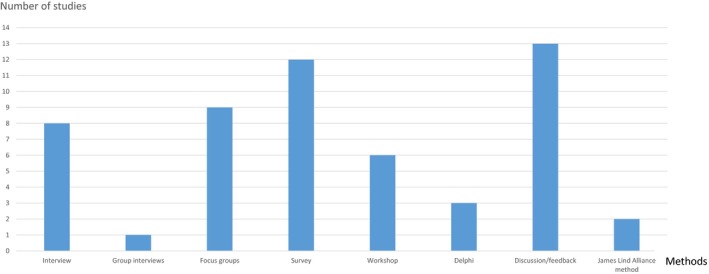
Methods applied for patient and public involvement

With regard to PPI methods, the studies included a range of various qualitative and quantitative scientific methods (Figure 3). PPI is also described in terms of processes that involved workshops, discussions and feedback sessions and where patients and carers participated in a variety of consultation, reference and expert groups. Finally, two studies use the JLA priority setting process,^27^, ^32^ which included the establishment of a steering group, surveys and consensus meetings between the assorted stakeholders.

### 3.3 Purposes and outcomes of PPI

All of the studies show alignment between the PPI purpose and the reported outcome. Most studies reflect the democratizing value of PPI in research. This is especially clear in studies that aim to identify and/or prioritize research topics that describe patient perspectives as essential to defining the future research agenda.^6^, ^25^, ^26^, ^27^, ^28^, ^32^, ^35^, ^43^, ^45^, ^49^

In studies designed to develop recruitment strategies, patients are involved to optimize and target recruitment, for example, to gain insight into patient opinions for clinical trial information materials,^47^ to develop more user‐friendly clinical trial websites,^44^ to define best time to recruit patients for patient‐reported outcome measures (PROM) research,^29^ to develop clinical trial decision aids^31^, ^40^ and to develop the study brand to increase recruitment and retention.^34^

When PPI is part of developing methods or entire study designs, patients and carers are involved to ensure, for instance, the relevance,^41^ population‐specific sensitivity,^46^ validity^50^ and ethics^37^ of the methods and study designs. Finally, the purpose of PPI at the dissemination stage is to ensure relevant education and information that can help reduce health disparities.^42^

### 3.4 PPI challenges and recommendations

During the review, we sought to extract specific PPI challenges and recommendations reported in the studies (Table 3). Challenges are not reported in all studies, but many of them described general methodological challenges and limitations, such as the issue of poor generalizability due to the limited number of participants, poor response rate^25^, ^43^, ^45^ or the qualitative design.^40^ Poor generalizability was also described in terms of the composition of the sample or included participants. Studies problematized the over‐representation of women^6^, ^42^ and the underrepresentation of ethnic minorities (n = 4),^6^, ^27^, ^42^, ^46^ and patients with an advanced and aggressive illness^6^, ^32^, ^48^ and newly diagnosed and relapsed patients.^43^ The issue of representativity reflects a classical methodological concern in studies that seek variation in their population but also touches upon the issue of opportunities to participate in PPI. In addition to this challenge, studies problematize the fact that PPI often involves the most socioeconomically advantaged patients who are already active in patient and consumer organizations^47^ and the difficulty of reaching and engaging less proactive patients^34^ and patients with a low level of health literacy.^49^

**Table 3:** Reported challenges and recommendations on patient and public involvement

Challenges	Text examples of the challenges	Recommendations	Text example of the recommendations
Limited patient skill level for engaging in the research process For example, Maintaining focus on research purpose (eg, prioritization)^25^ Understanding medical jargon^32^	“Patients spent most of the time in the groups talking about their own illnesses and how they had affected them and their families. Investigators brought discussions back to the issue of research priorities. For some groups patients were able to prioritise, for others patients wished to talk more about their own experiences.” (p.224)^25^ “Although attempts were made to formulate the research questions in lay language, in some situations it proved extremely difficult to do this in a concise fashion. This represented a major challenge for most patients and carers and, at the final workshop, many indicated they felt disadvantaged by their lack of understanding of the complex medical issues” (p.178)^32^	Build participant skills (the public and researchers)In terms of: Formal training 36, 39, 45 Holding separate meetings with researchers and patients^39^ Ensuring support from researchers^44^ Improve researchers ability to listen and respond to patient/public needs^44^	“Today's more informed society is eager and able to encourage a purposeful research culture and direction. Hence, we see the need to build and maintain a critical mass of researchers who are competent in partnering with consumer groups, which in turn can offer capable consumer representatives.” (p.7)^45^ “(…) training program for patient advocates was beneficial, as it prepared patient advocates to participate fully (eg, familiarizing them with the research process). Another step we found was to conduct separate meetings (…) to prepare them to participate in different steps in the study. For example (…) we met with patients prior to stakeholder Advisory Panel meeting to discuss patients’ presentations about their cancer experiences.” (p.101)^39^
Underrepresentation of certain groups For example, Ethnic minorities^6^, ^27^, ^42^, ^46^ Gender^42^ Age^27^ Socioeconomically disadvantages^47^ Less proactive patients^33^, ^49^ Advanced and aggressive illness^6^, ^32^, ^48^	“The lay participants predominantly identify as white and under 60 y old. It is notable that individuals of Asian (eg, people identifying as of Indian, Pakistani, Bangladeshi or other South Asian ethnicity) and Black ethnicity and older women who make up a substantial proportion of women diagnosed with EC are underrepresented” (p.143)^27^ “We acknowledge that this [PPI participants] is a group of self‐selected research aware young people (…). They voiced a concern on how to reach other young people who were less empowered and knowledgeable than themselves” (p.63)^33^	Consider sampling to achieve diversity	“More disadvantaged or ill patients would probably be less likely to participate in interviews and youth panels and more limited by the length of the questionnaire. In general, when involving patients in qualitative interviews, youth panels, and cognitive validation this limitation should be considered.” (p.175)^48^
Time‐consuming Requires extra resources^31^, ^36^, ^42^, ^50^	“Be prepared to spend time educating team members about the study process, and review as needed”… “Be prepared for a higher administrative burden that coincides with having a large team” (p.1115)^36^	Include time for meetings and planning/logistics^36^, ^42^	“Others who are considering such partnerships should ensure compensation for both academics and community members and that the academic's support include time for meetings and planning activities” (p.475)^42^
		Include extra costs forCare and comfort (support, rest, food)^50^ Financial support/compensation^39^, ^42^ Extra time and PPI expenditures^36^	“Special attention is required for adequate rest, nutrition, debriefing, and emotional support throughout the research process. We recommend that the basic (…) principles of respect, power sharing, and reciprocity, that researchers working with cancer population include in their research budgets resources to attend to issues and activities of care and comfort.” (p.245)^50^
		Involve patient/public participants as early as possible in the research process^39^	“One critical lesson is the importance of involving patients and other stakeholders as early as possible in the process. We began our collaboration (…) during the proposal funding stage, allowing everyone to have input in decisions about the study design” (p.101)^39^
		Ensure good relationships and clear roles and responsibilities Creating a respectful relationship, clear roles and well‐defined responsibilities^36^ Engaging in partnerships with well‐established networks and projects that were already a consumer priority^44^ Find “common language” to ensure effective communication between researchers and PPI participants^39^	“Before anything else, a relationship firmly grounded in trust and mutual respect must be established among key stakeholders. Academic researchers can earn trust and respect form CBOs (community‐based organizations) and their patients by asking about and responding to articulated needs. (…) CBOs can reap the benefits of incorporating research into their services by being receptive to the research process and acknowledging the value of gathering empirical evidence” (p.1114)^36^ “Roles and responsibilities were established early on in this study and outlined in a governance plan that was reviewed and approved by study leadership” (p.1114)^36^ “(…) critical success factors of this project were: (…) that the research team has worked with well‐recognised consumer groups with extensive networks (…)” (p.9)^44^

A few studies reflected on specific challenges regarding the involvement of patients and carers in the research process. One study revealed that the patients have difficulty focusing on research priorities as opposed to their own illness experiences.^25^ Another study stated that the medical jargon was difficult to understand and that patients found that prioritizing research issues was difficult because they were perceived as equally important.^32^ The PPI challenge mentioned most often was its time‐consuming nature and the surplus financial resources required.^31^, ^36^, ^42^, ^50^

In terms of recommendations, the majority of the studies (n = 19) did not have specific recommendations on the PPI process. However, PPI has implicit value and the studies recommended that engaging patients and carers in the research process is important for ethical and practical reasons. A few studies recommended specific methods, such as the value‐weighting approach as an acceptable and feasible method^43^; the community consultation model for ensuring a more ethical design^37^; focus groups as a valid method for formulating research ideas^25^; and the Delphi method for ensuring transparency and equity in the PPI process.^42^

The specific PPI recommendations included building participant competencies for PPI both among the patient/public participants and among the participating researchers.^45^ Improving patient/public competencies and skills could include formal training^36^, ^39^ and ensuring support from researchers^44^ and sensitive facilitation during the research process as it can be an upsetting experience to talk about illness experiences.^25^ Researchers can improve their ability to listen and respond to patient/public needs.^44^ Rush et al^36^ stressed the importance of creating a respectful relationship, clear roles and well‐defined responsibilities. Treiman et al^39^ recommended holding separate meetings with researchers and patients. Recommendations also included engaging in partnerships with well‐established networks and projects that were already a consumer priority.^44^ For process recommendations, one study recommended that patient and stakeholder involvement should be initiated early in the research process.^39^ Other process‐related recommendations were to include adequate time for meetings, planning^42^ and logistics.^36^ Some studies recommended including the following in the budget: care and comfort (support, rest, food),^50^ financial support/compensation,^39^, ^42^ cost of extra time and PPI expenditures.^36^ As described above, recommendations also involved taking representativity and the lack of diversity among PPI participants into consideration (see Table 3).

### 3.5 Quality assessment

Studies with a qualitative design were assessed according to CASP (n = 13) which was chosen because of its thorough examination of the quality of qualitative research (Appendix S1) and because it has been used in similar systematic reviews of PPI.^19^, ^20^, ^51^ The CASP assessment showed that the majority of the studies were of high quality. Studies that were of low quality did not apply the scientific methods generally applied in qualitative research (eg, expert panel discussions and workshops)^33^, ^42^ and did not fulfil the CASP criteria to the same degree as the other studies. One of the criteria that CASP assesses is whether the relationship between the researcher and participants has been critically examined. This includes researchers examining their own role, potential bias and influence on, for example, defining the research topic, data collection, recruitment and choice of location, but also how the researcher responded to events during the study and whether changes in the research design were considered. Despite the fact that this issue is a key aspect of PPI, many studies (n = 9) failed to meet the criteria. (Appendix S1)

The remaining quantitative descriptive and mixed methods studies (n = 14) were evaluated using MMAT, an instrument developed for mixed‐study reviews. We found variation in the quality assessments of the studies, where low quality assessments in the quantitative studies were related to issues regarding response rate. Low quality assessment of the qualitative studies mainly regarded the lack of description of researchers’ influence (in line with the CASP assessment of the qualitative studies). In both the MMAT and CASP assessment, low quality was often found in studies applying methods such as reference groups and other participatory research processes that were poorly described and therefore difficult to evaluate for quality (Appendix S1).

## 4 DISCUSSION

This review presented central aspects of PPI in cancer research based on an appraisal of relevant literature over the past decade to explore current trends and provide information for future PPI in cancer research. In the following, we summarize and discuss the findings by relating them to the broader PPI literature and point out implications for future PPI practice in cancer research.

### 4.1 PPI participants

Internationally, the frontrunners of PPI in cancer research continue to be the UK and the United States, followed by Australia, at least in terms of publications. This reflects the general trend of PPI activities^4^, ^19^ and is related to the strong organizational and policy foundation PPI has employed in these countries over a long period. The few publications (n = 5) originating from other countries indicated that PPI in cancer research outside the UK, the United States and Australia is still in its early stages.

Patient and public involvement population trends in cancer research have changed compared to the findings presented in earlier reviews,^5^, ^22^ where PPI activities predominantly took place in breast cancer research. The present review showed that a variety of diverse patient groups (when defined by cancer diagnosis) are involved in PPI activities. Moreover, cancer patients in palliative care^25^, ^28^, ^46^ and with short survival^32^ have been involved in research development, demonstrating that even patients at end of life are able and willing to participate. The review identified other ways to define PPI population groups in cancer research, for example, according to age^33^, ^34^, ^48^ or community/ethnicity.^36^ Our findings nonetheless call for paying critical attention to the composition of PPI participants, which currently in cancer research primarily continues to be well‐educated female participants from ethnic majority groups who are often already proactive in patient and community organizations. This point has been made and discussed in other reviews. Green found that certain groups (eg, youth, males and black and ethnic minorities) are often under‐represented in PPI in health research.^52^ However, Boote et al^1^ found that the population groups that were mostly involved in PPI in health research were black and minority ethnic groups followed by people with mental health problems, children and other “vulnerable” adults. While the findings by Boote et al demonstrate that PPI is practised in groups that in different ways may be categorized as vulnerable or marginalized, the findings in Green and the present review indicate that there is a lack of diversity among participants of PPI in other, for example, disease‐specific, research.

### 4.2 Research stages and methods

The review showed that studies have increasingly included PPI in the early stages of research. According to the earliest study included in the review, patient involvement in determining research priorities, especially in cancer research, was lacking and no comprehensive attempt was made to elicit patient views to inform the strategic direction of cancer research in the UK.^6^ Our review demonstrated that this aspect of PPI in cancer research has gradually grown in the last ten years; it has in fact become the research stage at which most PPI activities in cancer research take place internationally, especially in the UK. Although this is a positive development, our review shows that PPI is lacking in other research stages and that there are few examples of PPI being carried out at several research stages or throughout the entire research process. This finding is not isolated to cancer research, and similar findings have been found in other systematic reviews of PPI,^1^, ^13^, ^53^, ^54^, ^55^ which points out a central area for development of PPI in general.

An initial aim of our review was to make recommendations regarding methods and approaches to practising PPI across the research process. This has proven difficult for two reasons. First, PPI methods vary greatly and include scientific, process‐oriented and mixed approaches (eg, the JLA approach). We assessed the scientific quality of the studies according to CASP and MMAT and found that studies that have a high methodological quality, for example, do not necessarily report clearly on the PPI process. Similarly, studies whose methodological quality is low (due to a non‐scientific methodological approach) may describe the PPI process in greater detail. This puts into question the relevance of using appraisal tools developed for scientific methods when evaluating PPI studies. Another impediment to making recommendations based on experiences from other studies is the lack of critical reflection in the articles on the methods and PPI process applied. This is in line with other systematic reviews that have described the reporting of PPI as poor, inconsistent and lacking details on context, process, impact and conceptualization of PPI,^19^, ^20^, ^21^, ^56^, ^57^ which makes methodological recommendations difficult.^13^

The PPI literature addresses the challenge of synthesizing results from PPI articles to inform best practice, just as evaluation and reporting guidelines have been developed to support the reporting and comparability of PPI in research.^18^, ^51^, ^58^, ^59^ The recently updated version of the Guidance for Reporting Involvement of Patients and the Public (GRIPP2) comprises a short and long form. The latter can be used if reporting PPI is the primary objective of an article, while the former is useful for PPI as an integrative part of the research.^59^ The short form makes it possible to report on PPI while also reporting on the primary research objective based on conventional scientific criteria, as is the case with CASP and MMAT. The hope is that such tools will improve the comparability of future reporting on PPI, thereby allowing systematic reviews to inform best practice.

### 4.3 Purposes and outcomes of PPI

All of the included studies reported alignment between their specific purpose and outcome, pinpointing either the democratic value or research value, or both. The majority of the studies practised PPI at the research stage “Development of research focus,” which includes the definition and prioritization of research topics/questions. These studies clearly represented the democratic value of PPI, as expressed by van Merode et al^49^ “to formulate through a ‘bottom‐up’ approach original, relevant and realistic research goals, based on needs as conveyed by the patients”. However, since only a handful of studies reported that the prioritization of research has led to actual research projects, it is difficult to evaluate the democratic impact of PPI at this research stage. The prioritization of research has the potential to ensure patient/public partnerships and even lay‐leadership (cf. INVOLVE),^10^ but it depends on realizing the research priorities defined by patients/the public. If the research topics are not investigated, it can be argued that applying PPI to develop the research focus is a tokenistic activity.^9^ In relation to the discussion of the democratic value of recent PPI activities in cancer research, there are notably few examples of patient‐led research (Dear et al^44^ and van Merode et al^49^ are exceptions), which again reflects the general trend in PPI.^4^ Studies that examine how to enhance the quality of research predominantly focus on developing strategies to improve recruitment of study participants. The value of this purpose of PPI has been criticized for being too narrow.^60^

There are also studies that reflect both democratic and research‐oriented values in their stated purpose and outcomes. Studies focusing on method development argue that PPI ensures patient/public relevance and enhances validity in research. Similarly, studies involving patients/public throughout the research process also reflect democratic and research‐oriented values.^37^, ^38^, ^42^, ^50^ Islam et al,^38^ for example, demonstrated that the outcomes of PPI include a patient‐centred definition of outcomes, insight into research recruitment/retention and a relevant dissemination strategy. Chui et al^50^ found that the outcomes are increased validity, enhanced ethics and a high response rate.

By extracting the purpose and outcomes of PPI in the studies, we have attempted to identify the research‐oriented impact of PPI. However, the impact of PPI may also include the impact on the people involved (the patients/public, stakeholders, researchers), the wider community/community organizations and impact on implementation and change.^19^, ^21^ These kinds of impact have also been included in the GRIPP2 checklist^59^ to improve the reporting of PPI and thus the grounds for discussing and evaluating the impact of PPI. The recent year's debate about the impact of PPI has called for more detailed descriptions about the context and mechanisms of PPI to enhance our understanding of when, why and how it works.^21^, ^55^ It has been argued that the experiential knowledge of researchers and patients/the public is essential for understanding the complexity of the PPI collaboration and process and thus for the evaluation of the impact of different PPI approaches.^21^ In the present review, the majority of studies do not describe participants’ experiences or evaluation of their PPI participation and when provided, the descriptions are minimal.^25^, ^33^, ^38^, ^50^, ^61^

### 4.4 Challenges and recommendations

As described, most studies do not explicitly report challenges or recommendations on the PPI process based on their experiences. General methodological challenges and limitations are reported, but they do not specifically inform future PPI in cancer research. Likewise, general PPI recommendations, such as “we must involve patients in setting the research agenda”,^28^ or “Ideally consumers and researchers should always work together to identify and detail research topics”^45^ are also too vague to inform future PPI activities. The type of PPI challenges reported in other studies regarding, for example, conflicts among community concerns and research agendas and power relations between PPI participants and researchers^19^ were absent. This lack of critical attention is also identified in other systematic reviews of PPI, labelling it as a “publication bias”.^19^ This lack of critical discussion on the process and outcomes of PPI increases the risk of tokenism.

The studies in our review that more specifically address PPI challenges and recommendations confirm issues identified in other PPI reviews such as budgeting for extra costs and time spent,^13^, ^56^ as well as building competencies, skills and relationships,^54^, ^56^ and taking the composition of PPI participants into consideration, as discussed earlier. A handful of studies report specific challenges associated with involving patients with advanced and aggressive disease, where, for example, recruitment to panels and boards is difficult due to high patient mortality.^32^ Another specific recommendation for involving seriously ill people is to consider their special care needs, for example, being able to rest when participating in focus group discussions^50^ and ensuring sensitive facilitation during the research process, as it can be upsetting for the participating patient.^25^

### 4.5 PPI in cancer research and other medical fields

The findings of the present review describe the progress of PPI in cancer research within the last ten years, which demonstrates a continuous engagement in qualifying PPI. Compared to systematic reviews regarding PPI in other (medical) research areas, there are many similarities, as demonstrated in the discussion. There are, however, also additional insights that may have general relevance for PPI in other (medical) fields.

One of the findings the review shows that it is feasible and valuable to involve patients in research even when the survival rate is low and end of life near. This finding is particularly relevant for research in cancer and other life‐threatening diseases, but may also inspire the inclusion of patients who are in other ways perceived as being too vulnerable to participate in PPI. Another finding which has general relevance regards the composition of the participants in PPI activities, where the review shows that there is a growing critical awareness of the overrepresentation of certain groups, that is, women from socioeconomically advantaged and ethnic majority groups, who are often already proactive patients.

### 4.6 Implications for practice

The current trends identified in this systematic review point towards several areas that can be further developed within PPI in cancer research. Based on the findings of the review, we recommend that:

- PPI activities are expanded in countries besides the ones currently predominant in the field to generate international diversity in PPI in cancer research.
- Greater diversity is ensured in the composition of PPI participants regarding gender, ethnicity, health literacy, education and socioeconomically status.
- PPI activities go beyond the first stage of the research process “Development of research focus,” where PPI has become well established, to explore the potential of PPI more broadly throughout the research process.
- PPI reporting is qualified by including positive and negative experiences of the PPI process to inform future PPI, for example, by using GRIPP2.
- The special needs and preferences of seriously ill participants are considered to allow their participation in PPI activities.

### 4.7 Limitations

Our search strategy was limited to three scientific databases without grey literature searches. However, we were especially interested in reviewing peer‐reviewed PPI activities to ensure a standard for scientific quality.

It could be argued that it is a limitation that no patient or other public participant was involved in conducting the review. The reason for this is that the competencies needed to carry out a systematic review would require an introduction to the methodology and training, which was not feasible based on the scope of this review.

The assessment of the studies should be read with caution. As earlier discussed, some studies that are assessed to have a low standard of quality according to CASP and MMAT present thorough descriptions of the PPI process.

## 5 CONCLUSION

This systematic review described and discussed the current state of the international empirical literature on PPI in cancer research. PPI in cancer research has especially been integrated in the early stages of the research process, with most studies involving patients/the public in defining and prioritizing research. Involving patients/the public at this research stage is a strong democratic signal; however, if the research is not realized, PPI may risk becoming a token activity. As a result, we recommend that the reporting on PPI includes critical reflections regarding PPI methods and outcomes to avoid tokenism. Reporting on the positive and negative experiences of PPI will contribute to the further development of PPI and its potential.

## CONFLICT OF INTEREST

The authors declare that they have no conflict of interest.
