# Supplementary material for: Current trends in patient and public involvement in cancer research: A systematic review
Source: Health Expect. 2018 Oct 30;22(1):3–20. doi: 10.1111/hex.12841 (PMC6351419; doi:10.1111/hex.12841)
Supplement: Supplementary file 1 [file HEX-22-3-s001.pdf]

Appendix 1:
Quality assessment (CASP and MMAT)

CASP Appraisal questions	1 Aim	2 Method	3 Design-aim	4 Recruitment	5 Data collection	6 Researcher-participant relationship	7 Ethics	8 Analysis	9 Findings	10 Value	Notes
Ashley et al. 2012	yes	yes	yes	yes	yes	no	yes	yes	yes	yes	
Corner et al. 2007	yes	yes	yes	yes	yes	yes	yes	yes	yes	yes	
Ellis et al. 2012	yes	yes	yes	yes	yes	no	yes	yes	yes	yes	
Freysteinson 2010	yes	yes	yes	yes	yes	no	no	yes	yes	yes	Author states that it is a case study
Islam et al. 2014	yes	yes	yes	yes	yes	no	no	yes	yes	yes	
MacCarrier et al. 2016	yes	yes	yes	yes	yes	no	no	yes	yes	yes	Minimal description of ethics
Perkins et al. 2007	yes	yes	yes	yes	yes	yes	yes	yes	yes	yes	This study describes the qualitative study that led to the development of a questionmaire study (Perkins et al. 2008)
Rush et al. 2015	yes	CT	yes	CT	yes	yes	no	no	no	yes	
Sperling et al. 2017	yes	yes	yes	yes	yes	yes	yes	no	yes	no	
Taylor et al. 2015	yes	yes	yes	no	yes	no	yes	yes	yes	yes	Workshop related to Taylor 2016
Taylor et al. 2016	yes	yes	yes	no	yes	no	no	no	no	yes	Workshop - related to Taylor 2015
Treiman et al. 2016	yes	yes	yes	yes	yes	yes	yes	no	yes	yes	Patients are both data and partners
Wells et al. 2012	yes	yes	yes	yes	yes	no	yes	yes	yes	yes	

MMAT appraisal (mixed studies)	Screening questions		Qualitative studies (question 1.1-1.4)				Quantitative descriptive studies (question 4.1-4.4)				Notes
	Research objective	Design-objective	Data source	Analysis	Context	Researchers influence	Sample strategy	Sample representativity	Measurements	Response Rate	
Dellson et al. 2010	yes	yes	yes	yes	yes	no	yes	no	yes	yes	Participants are proactive patient advocates
Fleisher et al. 2014	yes	yes	yes	no	yes	yes	yes	no	yes	yes	
Clinton-Macharg et al. 2010	yes	yes					yes	yes	yes	no	
Moorcraft et al.2016	yes	yes					yes	yes	no	CT	Unclear who defined the 12 research areas that were prioritised. 780 patients participated/response rate unreported
Perkins et al. 2008	yes	yes					yes	yes	yes	CT	This article is related to Perkins et al. 2007 - focuses on survey results
Chiu et al. 2013	yes	yes	yes	yes	yes	yes	yes	yes	yes	yes	
Dear et al. 2011	yes	yes	CT	CT	no	no	no	CT	yes	no	Difficult to make appraisal, uses consumer reference group
van Merode et al.2016	yes	yes	Yes	no	yes	no	yes	yes	yes	no	
MacNair et al. 2016	yes	yes	Yes	yes	yes	yes	yes	yes	yes	yes	
Vargas et al. 2014	CT	CT	CT	CT	yes	CT	CT	CT	CT	CT	Difficult to make appraisal due to community partnered-participatory research process
Wan et al. 2016	yes	yes	Yes	yes	yes	no	yes	no	yes	CT	James Lind Alliance process
Vivat et al. 2012	yes	yes	Yes	yes	yes	CT	yes	yes	yes	CT	
Saunders et al. 2012	yes	yes	Yes	yes	yes	yes	yes	CT	yes	no	
Stephens et al. 2015	yes	yes					yes	CT	yes	no	James Lind Alliance process

Abbreviation:
CT= Cannot tell
